# A Care Transitions Electronic Clinical Pathway for Patients With Chronic Conditions: Qualitative Secondary Analysis

**DOI:** 10.2196/83235

**Published:** 2026-03-27

**Authors:** Joanne Roman Jones, Hongyi Wu, Christian J Tejeda, Allison B McCoy, Lipika Samal, Patricia C Dykes

**Affiliations:** 1 Manning College of Nursing and Health Sciences University of Massachusetts Boston Boston, MA United States; 2 Brigham and Women's Hospital Boston, MA United States; 3 Vanderbilt University Medical Center Nashville, TN United States; 4 Harvard Medical School Boston, MA United States

**Keywords:** care transitions, electronic clinical pathway, hospitals, multiple chronic conditions, patient education, primary care

## Abstract

**Background:**

Understanding patient educational requirements for care transitions is crucial for ensuring safe and effective care for patients with multiple chronic conditions (MCCs).

**Objective:**

Within a larger study developing an electronic clinical (e-clinical) pathway to support the safe transition for patients with MCCs from hospital to home and then to primary care, this study identified, evaluated, and integrated content to meet the educational requirements of potential end users of the e-clinical pathway. Participants in the primary study described preferred education content, education considerations, and education presentation and display.

**Methods:**

This study conducted a secondary qualitative analysis of transcripts from individual interviews (n=12 health care providers and staff) and focus groups (n=11 patient advisors) by using an inductive/deductive hybrid thematic analysis to explore the educational requirements of patients with MCCs for the e-clinical pathway.

**Results:**

We identified the following themes regarding specific education requirements for MCC transitions: (1) essential end-user education includes condition-specific, nutrition-specific, and medication-related content; (2) education must be truthful, accessible, and consider end-user health literacy; and (3) education presentation can be enhanced with graphics, reminders, and end-user resources.

**Conclusions:**

The findings were used to develop an educational pathway with targeted and tailored content to support patients with MCCs as they transition from acute hospitalization back to their primary care provider, which was implemented in the study app. Study findings contribute to a more nuanced understanding of end-user education requirements and informed enhancements to the patient-facing app used in the primary study.

## Introduction

Care transitions from hospital discharge to follow-up with primary care are critical moments for patients that involve the transfer of vital information [1]. This process requires close collaboration among hospital care teams, primary care providers, specialists, patients, and family or caregivers [2,3] to minimize the chance of miscommunication or information loss and to bridge potential gaps in care continuity. For patients with multiple chronic conditions (MCCs), the risks during this transition are particularly high, including an increased risk for hospital readmissions [4]. A 2018 analysis of the National Health Interview Study found that 27.2% of US adults had multiple (≥2) chronic conditions [5]. Patients with MCCs including diabetes mellitus (DM), congestive heart failure (CHF), and/or chronic kidney disease (CKD) face significant challenges as these and other comorbidities contribute to a higher disease burden and increased health care costs [6,7].

Understanding the patient educational requirements for care transitions is crucial for ensuring safe and effective care for patients with MCCs. For example, increasing services and touch points for high-risk populations can improve health care during critically important care transitions [8]. Digital health interventions that incorporate health education components are linked to greater user success [9]. In addition, effective patient education can enhance health literacy, which in turn supports self-management [10]. Research shows that mobile apps can improve health outcomes for individuals living with chronic illness [11], but a major challenge is the high dropout rate associated with app-based interventions [12]. Many older adults with MCCs and their caregivers express interest in using digital health portals, provided they offer features that patients find both useful and easy to navigate [13].

**Figure 1:**
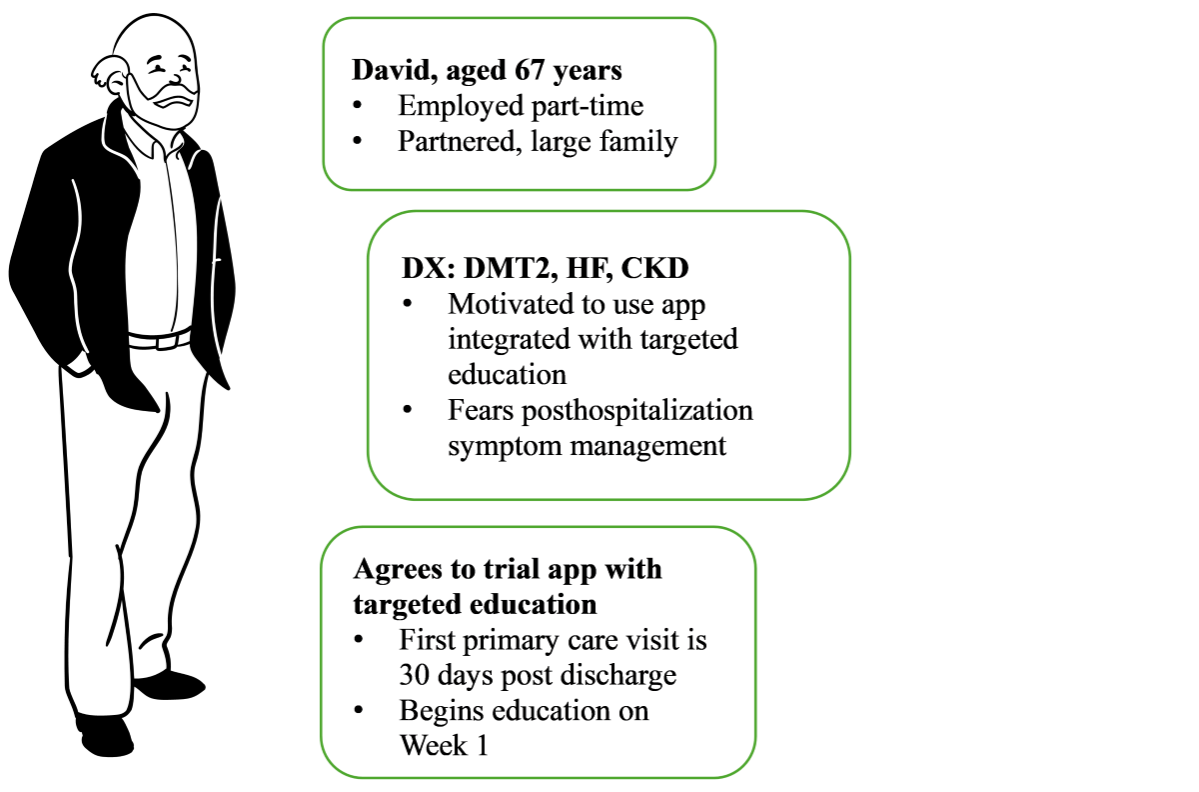
Sample persona of a potential intervention end user with multiple chronic conditions. CKD: chronic kidney disease; DMT2: type 2 diabetes mellitus; DX: diagnosis; HF: heart failure.

The electronic clinical (e-clinical) pathway configured within the MassGeneral Brigham patient portal specifically targets the transition from hospital to home for patients with MCC. This includes patients with varying combinations of DM, CKD, and CHF, or just one of these conditions along with another chronic illness (Figure 1). Previous work on the technical development of the e-clinical pathway has been described elsewhere [14]. In brief, the e-clinical pathway leverages Epic’s Care Companion module, which is an extension of the Epic MyChart patient portal [15]. The primary goal of the e-clinical pathway is to support patients in an outpatient setting and reduce the likelihood of readmissions. Epic provides a template that includes a selection of curated educational materials, and health care providers can customize and enhance this education as needed. Currently, Epic has not created a care plan specifically designed for discharged patients with MCCs to assist them and their caregivers during the transition from hospital to primary care.

**Figure 2:**
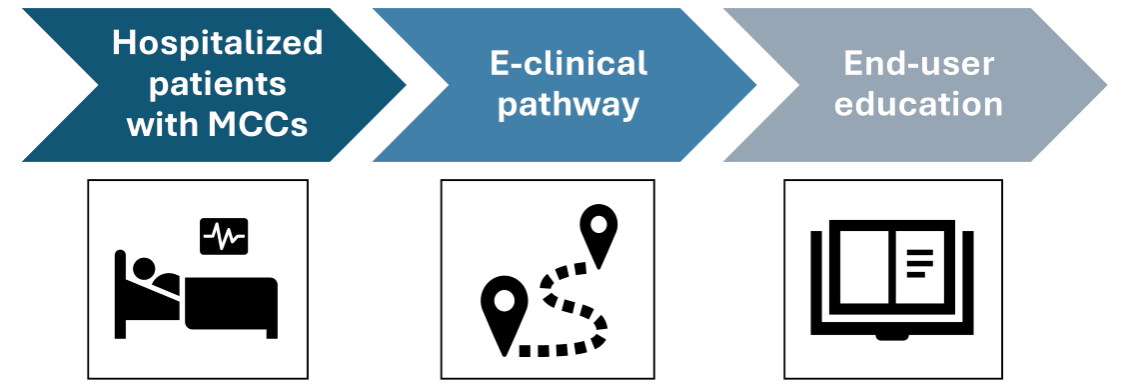
Educational content process. e-clinical: electronic clinical; MCCs: multiple chronic conditions.

This manuscript describes the process and key learnings from identifying, evaluating, and integrating educational content to address the needs of patients with MCCs and their caregivers: the end users of the e-clinical pathway. These efforts were guided by participant feedback from the primary study, which emphasized the need for information reinforcement (Figure 2) during the critical transition from acute hospitalization to home and ongoing care with their primary care provider. Thereafter, a secondary analysis of qualitative interview data explored the education needs of end users during the transition from acute care to home and primary care follow-up. To our knowledge, no publicly available content library currently addresses this specific care transition. This study aimed to identify, evaluate, and integrate content that meets these needs and to develop an education pathway that offers curated, personalized resources to support effective self-management at home.

The following were this study’s research questions:

- What educational requirements do patients with MCCs have during their transition from hospital to home and back to primary care?
- What educational materials will support patient self-management of their conditions and symptoms during the transition from acute hospitalization back to primary care?

## Methods

### Design

This was a qualitative secondary analysis of individual interviews and focus group data from a larger primary study that developed an electronic care transition tool to improve health outcomes of patients with MCCs using a stakeholder-centered design approach [14].

### Setting and Participants

The original primary study was conducted at Brigham and Women’s Hospital, a major academic medical center in New England, in the Northeastern United States. Health care providers and staff including hospitalists, outpatient physicians, physician assistants, nurse practitioners, nurses, and case managers were eligible to participate, as were patient advisors from the Brigham and Women's Hospital patient and family advisory council who were patients with MCCs who had been previously hospitalized. Purposive [16] recruitment occurred through email, including a detailed study purpose description. Health care providers and staff completed individual interviews, while patient advisors were randomly assigned to 1 of 3 focus groups for the ease, comfort, and schedules of the participants and to support open stakeholder communication with the study team.

### Data Collection and Analysis

For the primary study, the study team used a stakeholder-centered [17] design approach as the underlying conceptual basis for the planned research. The study team conducted both individual, virtual interviews involving multidisciplinary care providers and clinical staff, along with virtual focus groups involving patient advisors. Interviews and focus groups, conducted by multiple experienced team members and not exceeding 1 hour in length, continued until saturation [18] was reached. As part of the data collection in the primary study [14], participants openly shared and expounded on their views of educational content necessary for an effective electronic care transition tool. A secondary qualitative analysis was conducted to explore specific educational content research questions, yielding insights beyond the original scope of the primary study and addressing issues identified as important to the participants [19,20]. This report details this secondary qualitative analysis and the results. The methods used to collect the data used in the primary study have been published previously, as were the participant demographics [14]. Therefore, the purpose of this secondary analysis was to identify and explore the educational requirements of patients with MCCs for the e-clinical pathway as expressed by the participants in the primary study.

Interview guides, developed based on a literature review and reviewed by experts, were used for the focus groups and interviews, which were conducted with 12 health care providers and staff and 11 patient advisors in the primary study. The resulting transcripts, transcribed verbatim and anonymized, were the data corpus for this secondary qualitative analysis. Codes were generated both deductively from the interview guide and research questions using a template and framework matrix [21] and inductively from the primary study data, resulting in an inductive and deductive hybrid thematic analysis [22-24]. Three authors (CT, HW, and JRJ) performed the coding and deliberated the results. The remaining authors reviewed the analysis; discrepancies were discussed and resolved through team consensus. The analysis was iterative [25,26] and included team members to contribute to study reliability [27]. Throughout the process, regular team meetings and communications were conducted to calibrate data collection, to discuss findings, and to examine patterns and potential conclusions, encouraging team reflexivity [28,29]. The qualitative results were thereafter used to build an educational requirements Microsoft Excel spreadsheet, which was vetted by clinical experts and iteratively refined.

### Ethical Considerations

The Mass General Brigham Institutional Review Board (IRB) reviewed and approved all study activities (protocol 2022P001475) in addition to the University of Massachusetts Boston IRB (IRB ID 3476). Informed consent, including participants' ability to opt out, was received from all study participants before study participation. Resulting data were anonymized and deidentified, and strict confidence was maintained. All participants received a US $50 gift card at the end of study participation.

## Results

### Overview

A total of 23 individuals (n=12 clinical staff or primary care providers and n=11 patient advisors) participated in the original study; the majority were female (n=18) and White (n=19). The clinical staff or health care providers did not provide their ages; however, the ages of 10 of the 11 patient advisors ranged from 60 to 79 years. Participants in the original study described preferred education content, education considerations, and education presentation and display, and we identified through the qualitative secondary analysis the following themes regarding specific education requirements for MCC transitions: (1) essential end-user education includes condition-specific, nutrition-specific, and medication-related content; (2) education must be accurate, accessible, and consider end-user health literacy; and (3) education presentation can be enhanced with graphics, reminders, and end-user resources. The findings further resulted in a completed education pathway (Multimedia Appendix 1), with targeted and specific content for MCC transitions.

### Themes

#### Theme 1: Essential End-User Education Includes Condition-Specific, Nutrition, and Medication Content

Participants described frequently consuming online sources of medical information (“It’s my first go to,...I look at the medical websites” [Patient #2]) when searching out resources and references regarding their own medical conditions and drug interactions (“I do…reading on my own too.” [Patient #3]). Participants shared that patient education at discharge includes information about a new diagnosis or condition-specific information (“problem-based documentation…tailored to every single specific patient and their stay” [Clinician #6]). Condition-specific content is critical because, as 1 participant shared, “a lot of patients...just don’t understand their condition” (Clinician #2). Nutrition content is also important at discharge, including any specific “dietary restrictions” (Clinician #4). Dieticians routinely educate patients and caregivers (“anything about their diet” [Patient #4]) before hospital discharge. Medication changes, new medication information, or potential medication side effects are also critical education points for participants after discharge: “medication, education, and medication reconciliation,...that’s what the primary responsibility of the patient [is] when they are discharged from the hospital” (Clinician #6).

#### Theme 2: Education Must Be Accurate, Accessible, and Consider End-User Health Literacy

Potential patient end users want accurate education; participants shared that although they do reference medical information for their conditions online, at times internet sources “can raise a lot more questions” (Patient #2). Participants shared that they verify accuracy by confirming online information either with a trusted family member who is a clinician or another online source of information (“to validate what I read” [Patient #2]). For example, 1 patient-adviser participant shared that if “it’s something new or different or frightening I just pick up the phone and call my brother [the surgeon]” (Patient #3). Potential patient end users also want information that is accessible and “written clearly” (Patient #3). Participants appreciated the content of MedlinePlus [30] that specified “when to do what and...[that contained] enough information in plain language...so that people can refer to it. They’re not having to go elsewhere” (Patient #1).

However, and as one clinician participant explained, it is important to balance information requirements along with “the patient’s mental state [and] information overload” (Clinician #6), further sharing that the goal for discharge education is to provide “the most accurate and clear patient accessible information without overwhelming them with so much information...keeping it to the key points” (Clinician #6). Critically, patient education must consider “a patient’s health literacy so that when you are explaining these things, you’re not just assuming that they understand you” (Clinician #8). The education must also consider the potential patient end-user’s language of origin; for example, one patient participant shared: “one of the areas that I focus on a lot is...literacy...I think about the patient [who] doesn’t speak English perhaps, as a first language...and when I look at this in terms of multi syllable words and making an assumption that a patient can understand all of this. I see some opportunities. Maybe you could make this a little bit easier for those patients to follow” (Patient #5).

Participants indicated that education content intensity should vary by the individual patient need. As one clinician participant explained, every patient has “different ways of absorbing information” (Clinician #4). The study team configuring the e-clinical pathway presented 2 potential levels of education prepared for patients: one “standard” education pathway and one “superuser” education pathway. This would allow the app to push a smaller set of tasks to users that engage with tasks less frequently, including educational content. For example, superusers would receive 1 recommended reading per day; standard users would receive less content. Similarly, participants shared that potential end users may approach education content and intensity based on their individual background, needs, and comfort levels. For example, 1 participant shared that they would review content first on “the Brigham [website]...and then I’d look at Cleveland Clinic which I know is good for cardiac stuff...[but] that’s probably not the normal person” (Patient #2). In contrast, another participant indicated: “And I will read what’s on the Brigham...I mean I know nothing, so I have to presume what they’re...writing is accurate” (Patient #3).

#### Theme 3: Education Presentation Can Be Enhanced With Graphics, Reminders, and End-User Resources

The presentation of the education should integrate appealing graphics and/or visuals to enhance education understanding and readability. For example, one participant explained that they shared with the patient “a cup that we have in the hospital,...just to give them a visual...[to think of that much fluid]” (Clinician #5). Participants particularly appreciated the graphics on MedlinePlus (“some nice graphics” [Patient #1]; “really good graphics” [Patient #2]). Hospital discharge information similarly uses easily identifiable graphics: “stop signs and green lights and arrows. ...very clear and patient friendly” (Clinician #6). Education content can also be enhanced with reminders, including nutrition or diet reminders (“remember to stay away from extra salt” [Clinician #4]) or medication reminders. One participant shared: “I do find it very convenient to have that alarm...because regardless of age,...to help for compliance” (Patient #1). Another participant also suggested a digital health navigator (“if you had a helper” [Patient #2]) to support education and app use (“I would absolutely do it as long as I learned how” [Patient #2]).

### Sources of Education Content and Education Pathway Implications

As detailed in Table 1, the participant preferences expressed through the themes resulted in several implications for the sources of education content and the education pathway. The education content included medical condition–specific content for all 3 included conditions, with nutrition and medication content tailored to each condition. The primary education source was MedlinePlus, along with other reputable health websites, and all education links were structured and organized for end-user accessibility and health literacy.

**Table 1:** Secondary analysis findings and implications.

Constructs	Exemplar codes	Themes	Implications
Education content	Discharge topics include new medications and any medication changes Discharge teaching includes condition-specific nutrition and dietary information Education should be specific to the patient’s medical condition	Essential end user education includes condition-specific, nutrition, and medication content	Education included medical condition–specific content for all 3 included conditions Nutrition and medication content were tailored for each condition
Education considerations	Information should be accurate Education must consider end-user health literacy Education must be easily accessible	Education must be accurate, accessible, and consider end-user health literacy	Medline Plus was the primary education source, along with other reputable health websites All education links were structured for end-user accessibility and health literacy
Education presentation and display	Graphics can improve education understanding Reminders can support end-user needs Resources can support end-user uptake	Education presentation can be enhanced with graphics, reminders, and end-user resources	Education sources included visual aids and appropriate graphics to enhance education Reminders alert end-users to available education content Resources including a digital health navigator, with in-hospital support involving education access

The presentation of education included visual aids and graphics to enhance education; reminders to alert end users to available education content; and resources including a digital health navigator for end-user support (Table 1). These resulting implications were all directly addressed as the team developed and refined the education content pathway (“education pathway;” Multimedia Appendix 1).

The 3 conditions (and combinations of MCCs including DM, CHF, and CKD) were mapped onto an education pathway organized by postdischarge day and then curated, prioritized, and linked. Education will be pushed to the participant’s app interface, depending on the participant’s condition and postdischarge day, following the pathway. A section of the education pathway is excerpted in Table 2; the full spreadsheet of the final resulting education pathway is included in Multimedia Appendix 1, with live hyperlinks for freely available education content.

**Table 2:** Excerpt of the final education content pathway.

	Week 1				Week 2		
	Day 1	Day 2	Day 3	Day 1		Day 2	Day 3
Heart failure	Heart failure—discharge	Heart failure—medicine	Heart failure—home monitoring	Heart failure—overview		Heart failure—fluids and diuretics	Low salt diet
Diabetes	Diabetes type 2	Diabetes medicines	Diabetes type 2—meal planning	Managing your blood sugar		High blood sugar—self care	Low blood sugar—self care
Diabetes, CKD^a^, and HF^b^	Heart failure—discharge	Diabetes type 2	CKD (AKF^c^)	Heart failure—fluids and diuretics		Diabetes medicines	Keeping kidneys safe: smart choices about medicines (NIDDK^d^)

^a^CKD: chronic kidney disease.

^b^HF: heart failure.

^c^AKF: American Kidney Fund.

^d^NIDDK: National Institute of Diabetes and Digestive and Kidney Diseases.

**Figure 3:**
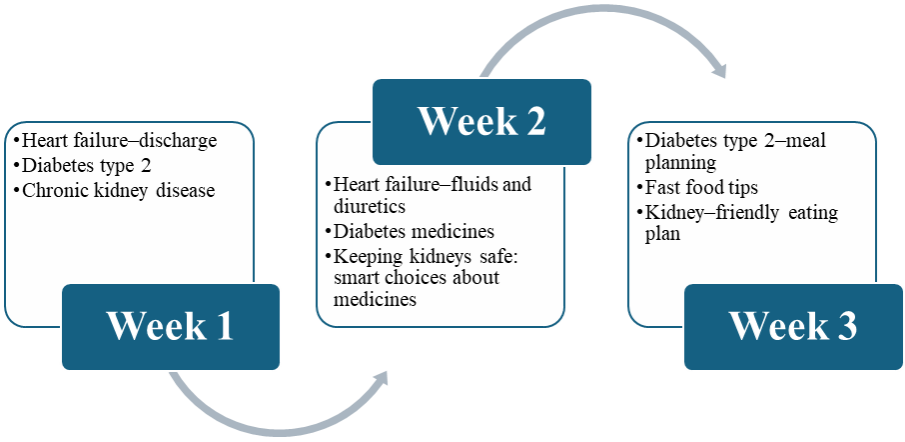
Weeks 1, 2, and 3 education topics for a multiple chronic conditions intervention end user with diabetes, chronic kidney disease, and heart failure.

While MedlinePlus [30] education sources make up most of the knowledge links, additional information was added to supplement CKD education, including information from the American Kidney Fund and the National Institute of Diabetes and Digestive and Kidney Diseases, which are also open source. As indicated in Table 2, several education content topics were included within each week, and this is further depicted in Figure 3. The article title is displayed as the display text, and these titles are hyperlinks directly linked to web pages (and live). Noted with more detail in the full education pathway in Multimedia Appendix 1 are the additional scripts that help orient the patient or user to the learning objective anticipated if the article link is clicked and read.

## Discussion

### Principal Findings

Through the secondary qualitative analysis of the data corpus, we identified necessary educational requirements of postdischarge information for patients with MCC, along with critical aspects of online patient education and, thereafter, transformed the findings into a targeted education content pathway. In sum, we have developed MCC care transition educational content that was integrated into our e-clinical pathway and application, also described in additional detail in a previous publication [14].

Barriers to self-efficacy for patients with chronic disease, including health literacy, access, and support, can be addressed with mobile apps and self-management programs [31,32]. Existing literature confirms the focus of this study and its findings, including the potential for apps to support self-management [33]. One randomized controlled trial of a personal health record mobile app found a maintained vs declining quality of care among participants who experienced the intervention [34]. Other literature has detailed how participants felt supported in their goal tracking through a mobile app [35] and reported increased motivation for behavior change [36]. As such, digital health technologies do have the potential to support patient outcomes. However, digital health technologies with targeted information for the education of patients with MCCs are key. Targeting self-management in the transition from hospital to home is critical in the education of patients with MCC. Despite the need, many educational resources, including online applications, only target one chronic illness, such as heart failure [37], or a specific self-management concept, such as fluid tracking [38]. Here, the team focused on providing comprehensive MCC educational content without contributing to cognitive overload. MedlinePlus [30] is the primary education source for the app education pathway and is a validated and free source of consumer health care educational content. In contrast to the available education from MedlinePlus for DM and CHF, unfortunately, detailed CKD information was insufficient for the established educational requirements. This is further illustrative of similar challenges with education scholarship for patients with CKD. For example, 1 review of available CKD apps found that nephrologists’ app evaluations indicated the health care providers perceived a lack in quality in the available content [39]. Another study evaluating CKD websites found that their evaluation justified the recommendation for content improvements [40]. Furthermore, existing literature also reflects the continued challenges with inadequate app use rates and insufficient engagement with digital health interventions [41,42], reflecting an ongoing tension with the app intentions and their ultimate success. We further note that although this study focused on the isolated component of education (within a larger MCC app with a core innovation of integration across the care continuum), the bidirectional data exchange between the app and the electronic health record system has the potential to enhance shared decision making [43] and information exchange, including among patients, caregivers, and health care providers. This potential remains a future area of interest and scholarship.

Additionally, the educational pathway is generalizable and reproducible in other health care systems. It is also potentially useful outside of health care institutions as a structure establishing an end-user guided process on a mobile app. The work and process of the study team and the resulting education pathway are detailed and included in this manuscript specifically so that other health care systems, or others, can freely replicate the process. This information provided could be used by another facility to adjust the e-clinical pathway for their own needs, based on the description of the process in this manuscript and other related manuscripts [14], minimizing process burden and cost. The team used content in the public domain, which is generalizable as this content can be easily adopted and used in low resource settings. Stakeholder involvement in the educational requirement process was intended to increase potential uptake and dissemination [44]; disseminating knowledge increases the potential for impact [45]. Although other educational sources were considered, often with many benefits, including curated and organized content, one critical aim was to make the education pathway a process that could be replicated. As such, the study team decided to primarily use MedlinePlus [30] for the educational content, along with open-source CKD education from the American Kidney Fund and the National Institute of Diabetes and Digestive and Kidney Diseases. While MedlinePlus [30] is an exceptional, well-sourced, federally backed, and free education source, there were aspects of this education source that were oftentimes unwieldy, expansive, and not limited to care transitions information. Future work should consider positioning analysis within a framework of integrated care [46] or collaborative care models [47] to emphasize system-level requirements for cross-sector information flow and accountability.

Finally, the importance of accessible language, digital health navigators, and family involvement evident in the results also indicates concordance with the existing literature. Adult health literacy proficiency remains low and mobile apps focused on language and end-user accessibility can support aspects of health literacy, including health information seeking [48]. Participants in this study expressed their desire for robust support, such as through a digital navigator, and this is reflected in the available science expounding on the potential positive impact of digital health navigators on patient app use [49]. Participants also brought up the positive impact of family involvement in their own digital health technology experiences; existing scholarship confirms that family engagement and support can facilitate patients’ adoption of a digital health solution [50], although further studies should also consider the digital literacy of caregivers as a complementary identified need [51]. In sum, future work involving mobile apps and other digital health technologies should remain mindful of the many barriers to participant recruitment and technology use, although our results suggest patient and family engagement, digital health navigator involvement, and language accessibility may be modifiable and successful factors to consider.

### Limitations

This work has some limitations that should be noted. First, this study was conducted among participants very engaged with health care services and knowledgeable about the postdischarge process. However, this study intentionally and purposefully included early stakeholder involvement, extensive health care provider and patient representative reviews and feedback, and iterative work with the study team and experts to succinctly create targeted and essential educational content. Second, the secondary qualitative analysis of educational requirements was conducted before app completion and testing. Future work will be needed to confirm and validate educational content fit with app end-user needs and larger primary study aims. Future work will also need to consider sustainability of the app overall, including maintenance of education content and user feedback. Third, the secondary qualitative analysis methodology of the original dataset inherently constrains a full understanding of e-care team coordination and interprofessional perspectives. Future scholarship directly addressing these critical perspectives is needed.

### Conclusions

This study effectively produced, organized, and vetted the educational requirements to support a patient with MCCs during the critical posthospitalization transition period. Our secondary qualitative analysis extended the work of the primary study with a conceptual focus on patient and caregiver educational requirements. Study findings support a deeper understanding of end-user education requirements and further enhanced the resulting education resources intended for the app in the primary study. The end-user educational requirements and the included resulting education pathway implemented in the study app are freely available to support future research, implementation, and evaluation efforts (including, potentially, contextual tailoring, scalability, and impact testing) aimed at improving care transitions for individuals with MCCs.

## Data Availability

The resulting education pathway table is provided as Multimedia Appendix 1, together with the manuscript. In addition, the datasets generated or analyzed during this study are available from the corresponding author upon reasonable request.

## Appendix

Multimedia Appendix 1 Completed education pathway.

## Abbreviations

CHF: congestive heart failure
CKD: chronic kidney disease
DM: diabetes mellitus
e-clinical: electronic clinical
IRB: institutional review board
MCCs: multiple chronic conditions
