# Supplementary material for: A Care Transitions Electronic Clinical Pathway for Patients With Chronic Conditions: Qualitative Secondary Analysis
Source: J Particip Med. 2026 Mar 27;18:e83235. doi: 10.2196/83235 (PMC13069371; doi:10.2196/83235)
Supplement: Multimedia Appendix 1 [file jopm_v18i1e83235_app1.pdf]

| | A | B | C | D | E | F | G | H | I | J | K | L | M |
|---|---|---|---|---|---|---|---|---|---|---|---|---|---|
| 1 | | Week 1 | Week 1 | Week 1 | Week 2 | Week 2 | Week 2 | Week 3 | Week 3 | Week 3 | Week 4 | Week 4 | Week 4 |
| 2 | Heart Failure (use the article title as Handout Display Text) | Heart failure - discharge | Heart failure - medicine | Heart failure - home monitoring | Heart failure overview | Heart failure - fluids and diuretics | Low-salt diet | Fast food tips | How Can I Live with Heart Failure? | Lifestyle Changes for Heart Failure | Mediterranean diet | Dietary fats explained | Heart failure - what to ask your doctor |
| 3 | Patient Display Name | Learn how to manage your heart failure after hospital discharge | Learn the importance of your heart failiure medication | Learn how to manage your heart failure at home | Learn about heart failiure | Learn why it's important to watch your fluid intake | Learn what to eat to keep your heart healthy-1 | Learn what to eat to keep your heart healthy-2 | Learn what lifestyle changes can help you manage heart failure and improve your quality of life | Learn how to improve your quality of life living with heart failure | Learn what to eat to keep your heart healthy-3 | Learn what to eat to keep your heart healthy-4 | Learn what to ask your doctor about heart failure |
| 4 | Patient Description (Learning Objective) | By reading this article, you'll gain a better understanding of the self-care steps to take at home regarding diet, fluid intake, activity, monitoring symptoms, and taking medications for heart failure. | This article will help you understand why you're taking your heart failure medication. | This article will help you identify warning signs indicating worsening heart failure. | This article will help you understand the symptoms, diagnosis, and treatment options for heart failure. | This article will help you understand the importance of avoiding fluid buildup and limiting fluid intake when you have heart failure. | This article will help you understand the importance of limiting sodium intake. | This article will help you understand how occasional fast food meals can still fit into an overall balanced diet. | This article will help you understand how to make essential lifestyle changes for effectively managing heart failure. | This article will help you understand small, positive lifestyle changes that can be made to improve the quality of life for those living with heart failure. | This article will help you understand the importance of eating plant-based foods and healthy fats. | This article will help you understand how some types of fat are healthier than others. | By reading this article, you will gain a better understanding of the how to make the most of your follow-up visit and ask your provider the appropriate questions. |
| 5 | Diabetes | Diabetes Type 2 | Diabetes Medicines | Diabetes type 2 - meal planning | Managing your blood sugar | High blood sugar - self-care | Low blood sugar - self-care | Diabetes - preventing heart attack and stroke | Diabetic Diet | Diabetes - keeping active | Snacking when you have diabetes | Diabetes - when you are sick | Type 2 diabetes - what to ask your doctor |
| 6 | Patient Display Name | | | | | | | | | | | | |
| 7 | CKD (NIDDK&AKF) | Chronic kidney disease (CKD) (AKF) | Keeping Kidneys Safe: Smart Choices about Medicines (NIDDK) | Managing Chronic Kidney Disease (NIDDK) | Healthy eating & activity (AKF) | How can CKD affect my day-to-day life? (NIDDK) | Tests for kidney disease (AKF) | Stages of kidney disease (AKF) | Kidney-friendly eating plan (AKF) | Health problems caused by kidney disease (AKF) | Your CKD toolkit (AKF) | Kidney-friendly holidays (AKF) | eating right for CKD (NIDDK) |

| | A | B | C | D | E | F | G | H | I | J | K | L | M |
|---|---|---|---|---|---|---|---|---|---|---|---|---|---|
| 8 | Diabetes & CKD | Diabetes Type 2 | Chronic kidney disease (CKD) (AKF) | Diabetes Medicines | Keeping Kidneys Safe: Smart Choices about Medicines (NIDDK) | Diabetes and kidney disease | Diabetic Kidney Problems | Diabetes type 2 - meal planning | Kidney-friendly eating plan (AKF) | Health problems caused by kidney disease (AKF) | Fast food tips | Managing your blood sugar | Managing Chronic Kidney Disease (NIDDK) |
| 9 | HF & CKD | Heart failure - discharge | Chronic kidney disease (CKD) (AKF) | Heart failure - medicines | Keeping Kidneys Safe: Smart Choices about Medicines (NIDDK) | Heart failure - fluids and diuretics | Managing Chronic Kidney Disease (NIDDK) | Heart failure - home monitoring | Low-salt diet | Kidney-friendly eating plan (AKF) | Stages of kidney disease (AKF) | Fast food tips | Heart failure - what to ask your doctor |
| 10 | Diabetes & HF | Heart failure - discharge | Diabetes Type 2 | Heart failure - fluids and diuretics | Diabetes Medicines | Diabetes - preventing heart attack and stroke | Fast food tips | Diabetes type 2 - meal planning | Diabetes - keeping active | low-salt diet | How Can I Live with Heart Failure? | Snacking when you have diabetes | Managing your blood sugar |
| 11 | Diabetes & CKD & HF | Heart failure - discharge | Diabetes Type 2 | Chronic kidney disease (CKD) (AKF) | Heart failure - fluids and diuretics | Diabetes Medicines | Keeping Kidneys Safe: Smart Choices about Medicines (NIDDK) | Diabetes type 2 - meal planning | Fast food tips | Kidney-friendly eating plan (AKF) | Low-salt diet | Snacking when you have diabetes | Diabetes - keeping active |
| 12 | | | | | | | | | | | | | |
| 13 | Exercise | Exercise and age | Day 1, 6, 10, 14, 18, 22, 26 Sitting exercise handout | | | | | | | | | | |
| 14 | | Physical activity | | | | | | | | | | | |
| 15 | | Exercise and physical fitness | | | | | | | | | | | |
| 16 | | | | | | | | | | | | | |
| 17 | Nutrition | Day 4: Myths about nutrition and physical activity | Day 8: low-salt diet | Day 12: Fast food tips | Day 16: Understanding the DASH diet | Day 20: Nutrition | Day 24: Dietary fats explained | Day 28: Mediterranean diet | | | | | |
